# Multiple intraventricular metastases from lung adenocarcinoma with EGFR G719X mutation: a case report

**DOI:** 10.1186/s12890-020-1168-0

**Published:** 2020-05-11

**Authors:** Chen Kong, Dan Zhou, Ning Wu, Chong Bai

**Affiliations:** Department of Respiratory and Critical Care Medicine, Changhai Hospital, Second Military Medical University, Shanghai, China

**Keywords:** Case report, Intraventricular metastases, Lung adenocarcinoma, Tyrosine kinase inhibitor

## Abstract

**Background:**

Brain is one of the most common target organ of lung cancer metastasis, while descriptions of intraventricular carcinomatosis could hardly be found among previous cases. To date no cases from lung adenocarcinoma have been reported in the literature.

**Case presentation:**

We report here a case of multiple intraventricular metastases from lung adenocarcinoma with EGFR G719X mutation. This 64-year-old woman was referred to our hospital with complaints of dizziness and vomiting. Target therapy with afatinib was initiated and the lesions in both lung and brain achieved good partial responses.

**Conclusions:**

This case report revealed a phenomenon of rare intraventricular metastasis from lung cancer, which should be carefully distinguished from primary ventricular tumors. Compared to brain parenchyma metastasis, intraventricular lesions would cause more severe symptoms which may be rapidly progressive. Target therapy could become a potential option in such patients with non-drugresistant EGFR mutations.

## Background

Up to 50% of pulmonary carcinoma patients would develop brain metastases throughout their clinical courses, and 10–25% of them have brain metastases at the time of initial diagnosis [1–3]. Adenocarcinoma is the most common subtype of lung cancer in women. Although lung cancer can theoretically disseminate to ventricles, no case of isolated intraventricular metastasis of lung adenocarcinoma has been reported in the literature. Here, we present this unique case of a Chinese woman who was confirmed as late stage lung adenocarcinoma with intraventricular metastases without parenchyma lesions and had a good response to target therapy with Epidermal Growth Factor Receptor- tyrosine kinase inhibitor (EGFR-TKI).

## Case presentation

A sixty-four-year-old Chinese woman, with a ten-year history of diabetes and hypertension, presented with serious dizziness and vomiting in a month.

**Fig. 1:**
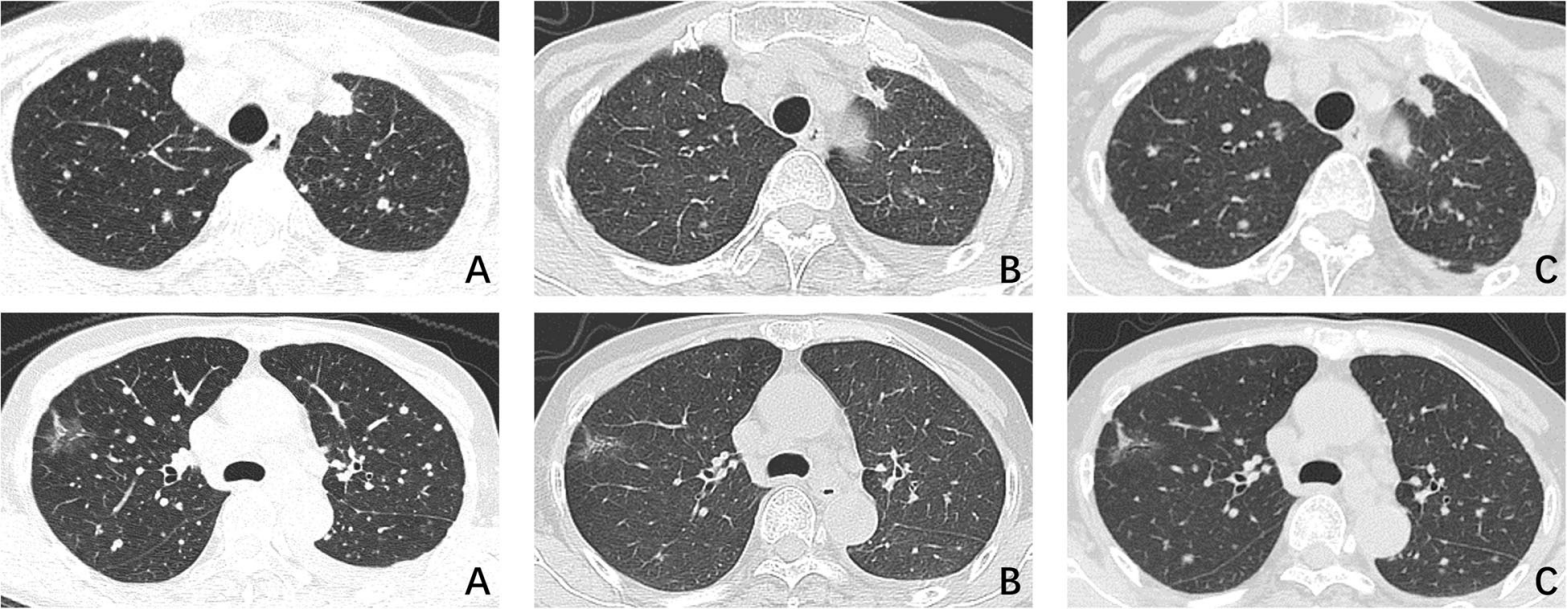
CT scans of chest during different period of treatment **a** Chest CT scan showed a 1 cm × 1 cm irregular nodule in the upper lobe of left lung, with diffuse bilateral pulmonary metastases. **b** Bilateral nodules evidently shrunk after treatment with afitinib for 3 months. **c** Lesions of lung progressed after ten months of targeted therapy

**Fig. 2:**
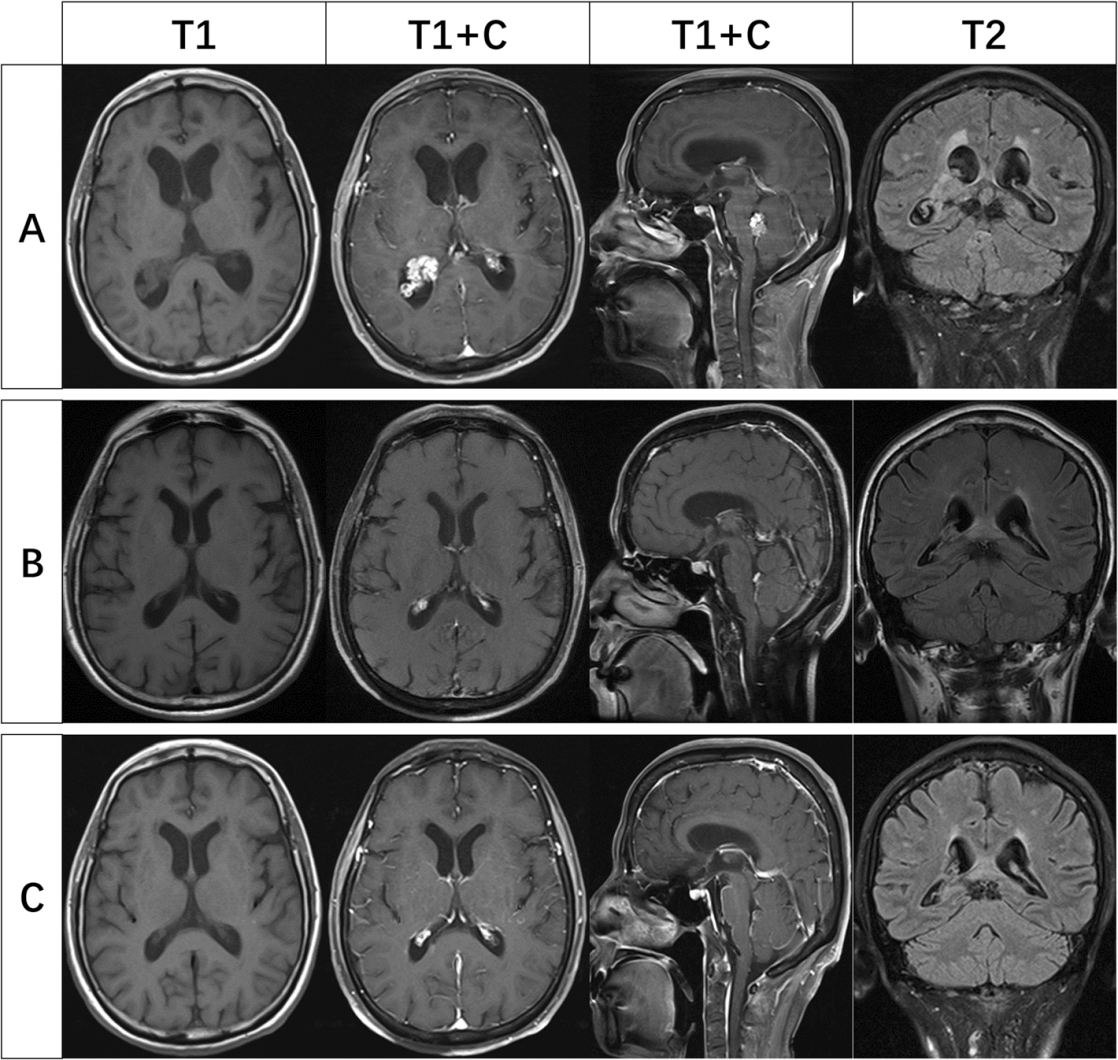
MRI of brain during different periods of treatment **a** Contrast-enhanced MRI of brain showed heterogeneously enhancing lesions at the posterior horn of the right lateral ventricle and the IV ventricle, with upper ventricle hydrocephalus and edema of the surrounding parenchyma. T2FLAIR showed slightly higher signal of the masses. **b** Intraventricular lesions and surrounding area of edema observably shrunk with restored ventricular dilatation after 3-month treatment with afitinib. **c** Masses in the brain remain stable after 10-month treatment while lesions of lung progressed

Positron emission tomography (PET) performed in October 2017 indicated a nodular lesion in left upper lung with diffuse bilateral lung metastases (Fig. 1a) and multiple intraventricular masses. Magnetic resonance imaging (MRI) of brain showed a mass at the posterior horn of the right lateral ventricle and the IV ventricle, respectively. T1 brain MRI sequences showed isointense lesions with heterogeneous enhancement on contrast and T2 brain MRI sequences showed hyperintense masses, remarkable dilation and hydrocephalus of upper cerebral ventricle and parenchyma edema around encephalocoele. No obvious involvement of brain parenchyma was found (Fig. 2a).

Lung adenocarcinoma was histopathologically diagnosed by endobronchial ultrasound transbronchial needle aspiration (EBUS-TBNA). Afatinib, a second generation Epidermal Growth Factor Receptor- tyrosine kinase inhibitor (EGFR-TKI) was administered at a dose of 30 mg qd due to EGFR mutation in exon 18 (G719X). The symptoms of the patient improved significantly within one month and most of the daily activities were affordable. Reevaluation after three-month treatment showed partial remission in both lung and brain lesions (Figs. 1b and 2b). Repeated computed tomography (CT) of chest after ten months’ treatment in September 2018 showed bilateral pulmonary nodules increased and enlarged, while intracerebral lesions remained stable (Figs. 1c and 2c). Severe headache and dizziness reoccurred half a month later and the symptoms aggravated rapidly afterwards. The patient eventually died with an overall survival of 12 months.

## Discussion and conclusions

Intraventricular neoplasms account for less than 1% of intracranial neoplasms [4], which were mostly described in primary central nervous system malignant tumors, such as choroid plexus papilloma, carcinoma, ependymoma, subependymoma, subependymal giant cell astrocytoma, central neurocytoma and meningioma. Metastasis shares about 2% of all intraventricular neoplasms [5]. Intraventricular metastasis from a carcinoma occurs even rare (0.9–4.6% of all brain metastases) [6]. The most common intraventricular metastases from epithelial malignancies include renal [5, 6], lung [1], colon carcinomas and melanoma.

It has been widely recognized that brain metastases frequently occur in the early course of lung cancer, especially in small cell lung cancer (SCLC) [7]. Among all the brain metastases, parenchymal metastasis is the most common way, whereas its prognosis is poor. Intraventricular metastasis from lung cancer was rarely reported. Surgical management of lateral-ventricle metastases at the University of Texas M. D. Anderson Cancer Center was reported in 2010 [4]. Among the 29 cases reported, 22 had single or multiple intraventricular lesions without synchronous intraparenchymal metastases and only 2 of them were diagnosed as lung cancer. Esther Una [8] reported the first case of intraventricular metastasis developed from a man with SCLC in 2012. Another case of intraventricular metastases from SCLC presented with irregular thickening around edges of lateral ventricles and fourth ventricle in MRI images was reported by Hao Chen [9]. Saraj K. Singh [10] introduced a case of pulmonary squamous cell carcinoma with intraventricular metastases which had been misdiagnosed as meningioma before surgery. In our case, unique metastatic form of bulky masses in ventricles without involvement of brain parenchyma was shown in a patient with lung adenocarcinoma.

The lateral ventricles are the most common sites of intraventricular metastases due to the abundant vascular supply of choroid plexus [11]. It is difficult to distinguish intraventricular metastases from primary central nervous system tumors, especially choroid plexus papilloma and meningioma, given the brain MRI screenings merely. Choroid plexus papilloma is a slow-growing benign tumor originated from choroid plexus epithelial cells in ventricles, which is more common in children under 10 years old. It predominantly locates in the lateral ventricles and the fourth ventricle, 50 and 40% respectively, with potential of malignant transformation into choroid plexus carcinoma. Typical imaging findings consist of clear boundary of mass with equal or low T1WI, equal or slightly higher T2WI. Choroid plexus tumors are able to secrete plenty of cerebrospinal fluid, which cause obstructive or communicating hydrocephalus, resulting in the symptoms of intracranial hypertension such as dizziness and vomiting. Surgical resection is the only proven treatment. In respect to meningioma, the peak age range of it is 30–60 years [11]. Most meningiomas are found in the atria of the lateral ventricles and less commonly in the third and fourth ventricles [12]. On MRI, lesions are usually well-defined, iso- to hypointense on T1WI, and iso- to hyperintense on T2WI. In this case, the elder female patient does not belong to the high incidence group of these primary central nervous system diseases. Despite the overlap of imaging findings with these diseases, metastasis is the first diagnosis to be considered due to the underlying pulmonary malignancy.

Afatinib, a second generation EGFR-TKI, was approved by the U.S. FDA for metastatic NSCLC with uncommon non-drugresistant EGFR mutations, such as L861Q, G719X, and S768I. Afatinib can achieve half maximal inhibitory concentration (IC50) against EGFR mutation in the cerebrospinal fluid [13] so that theoretically it can create an inhibitory effect on intraventricular metastases from lung cancer with minimal adverse effects. Molecular analysis indicated EGFR mutation (G719X) in this patient and both lesions in bilateral lungs and brain showed significant improvement after treatment with afatinib, which supported that the intraventricular lesions were metastatic disseminations of lung adenocarcinoma. However, it is a pity that on account of the severe dizziness and vomiting, the patient refused to accept lumbar puncture or surgery for intracranial histological tests.

The described case adds pulmonary adenocarcinoma to the possible origin of an intraventricular cerebral metastasis. Furthermore, whether the patient has brain metastases or not, afatinib may be a potential option for patient with uncommon mutations of EGFR.

## Data Availability

The datasets used and/or analyzed during the current study are available from the corresponding author on reasonable request.

## Abbreviations

MRI: Magnetic Resonance Imaging
EBUS-TBNA: Endobronchial Ultrasound Transbronchial Needle Aspiration
TKI: Tyrosine Kinase Inhibitor
EGFR: Growth Factor Receptor
PET: Positron Emission Tomography
CT: Computed Tomography
SCLC: Small Cell Lung Cancer
IC50: Half Maximal Inhibitory Concentration

## References

[1] Ulahannan D, Khalifa J, Faivre-Finn C, Lee SM (2017). Emerging treatment paradigms for brain metastasis in non-small-cell lung cancer: an overview of the current landscape and challenges ahead. Ann Oncol.

[2] Schouten Leo J, Joost R, Huveneers Hans AM (2002). Incidence of brain metastases in a cohort of patients with carcinoma of the breast, colon, kidney, and lung and melanoma. Cancer..

[3] Barnholtz-Sloan Jill S, Sloan Andrew E, Davis Faith G (2004). Incidence proportions of brain metastases in patients diagnosed (1973 to 2001) in the metropolitan Detroit Cancer surveillance system. J Clin Oncol.

[4] Hassaneen W, Suki D, Salaskar AL (2010). Surgical management of lateral-ventricle metastases: report of 29 cases in a single-institution experience. J Neurosurg.

[5] Sava I, Sava A, Şapte E (2013). Intraventricular metastatic clear cell renal carcinoma. Romanian J Morphol Embryol.

[6] Raila FA, Bottoms WT, Fratkin JD (1998). Solitary choroid plexus metastasis from a renal cell carcinoma. South Med J.

[7] Aizer Ayal A, Lee EQ (2018). Brain Metastases. Neurol Clin.

[8] Uña E. Intraventricular metastases from small cell carcinoma of the lung. BMJ Case Rep. 2012. 10.1136/bcr.12.2011.5440. 10.1136/bcr.12.2011.5440 PMC3351662 22605865

[9] Chen H, Raza HK, Shi HJ, et al. A rare case of small cell carcinoma of lung with intraventricular metastasis. Br J Neurosurg. 2017. 10.1080/02688697.2017.1327020. 10.1080/02688697.2017.1327020 28497995

[10] Singh SK, Agarwal H, Singh P (2018). Intraventricular metastasis mimicking meningioma. Surg Neurol Int.

[11] Smith AB, Smirniotopoulos JG, Horkanyne-Szakaly I (2013). From the radiologic pathology archives: intraventricular neoplasms: radiologic-pathologic correlation. Radiographics.

[12] Muly S, Liu S, Lee R (2018). MRI of intracranial intraventricular lesions. Clin Imaging.

[13] Ma CH, Huang CJ, Tang DJ (2019). Afatinib for advanced non-small cell lung Cancer in a case with an uncommon epidermal growth factor receptor mutation (G719A) identified in the cerebrospinal fluid. Front Oncol.

